# Effect of LED Lighting on Physical Environment and Microenvironment on In Vitro Plant Growth and Morphogenesis: The Need to Standardize Lighting Conditions and Their Description

**DOI:** 10.3390/plants11010060

**Published:** 2021-12-25

**Authors:** Araceli Barceló-Muñoz, Marta Barceló-Muñoz, Alfonso Gago-Calderon

**Affiliations:** 1 IFAPA—Centro de Málaga, Cortijo de la Cruz s/n, 29140 Malaga, Spain; marta.barcelo@juntadeandalucia.es; 2 Proyectos de Ingeniería, Departamento de Expresión Gráfica Diseño y Proyectos, Universidad de Malaga, 29071 Málaga, Spain

**Keywords:** light-emitting diodes (LEDs), Grolux fluorescent lamps, light spectral characterization, in vitro culture, plant tissue culture, temperature, in vitro environmental conditions

## Abstract

In the last decades, lighting installations in plant tissue culture have generally been renewed or designed based on LED technology. Thanks to this, many different light quality advances are available but, with their massive implementation, the same issue is occurring as in the 1960s with the appearance of the Grolux (Sylvania) fluorescent tubes: there is a lack of a methodological standardization of lighting. This review analyzes the main parameters and variables that must be taken into account in the design of LED-based systems, and how these need to be described and quantified in order to homogenize and standardize the experimental conditions to obtain reproducible and comparable results and conclusions. We have designed an experimental system in which the values of the physical environment and microenvironment conditions and the behavior of plant tissue cultures maintained in cabins illuminated with two lighting designs can be compared. Grolux tubes are compared with a combination of monochromatic LED lamps calibrated to provide a spectral emission, and light irradiance values similar to those generated by the previous discharge lamps, achieving in both cases wide uniformity of radiation conditions on the shelves of the culture cabins. This study can help to understand whether it is possible to use LEDs as one standard lighting source in plant tissue culture without affecting the development of the cultures maintained with the previously regulated protocols in the different laboratories. Finally, the results presented from this caparison indicate how temperature is one of the main factors that is affected by the chosen light source.

## 1. Introduction

Until recently, white light (in cool, daylight, neutral or warm versions) or Grolux fluorescent lamps were used almost universally to supply light in in vitro growth rooms, among other reasons because of the uniform light intensity they emit. Nowadays, these fluorescents have given way to LED luminaires that, among many other options, allow us to choose the specific wavelengths we want to send to the plants depending, for example, on the species or process of interest.

In the following chapters, a technological review of fluorescent and LED lamps is presented from a general point of view and, more specifically, in terms of their possibilities related to in vitro culture. Next, a review of recently published studies that include the use of different alternatives with LEDs on in vitro plant tissue culture will be considered, focusing on the lighting variables of the installations used in each case which have more relevance on the plants. The outcome of this review will be used as the basis to establish appropriate characterization parameters of these light settings, highlighting the advantages and drawbacks of the different alternatives found, and their critical analysis.

Finally, an experimental setup with Grolux and a LED lighting system calibrated to offer a similar spectral emission was examined. This generated a controlled environment in which to apply the results of the previous characterization protocol and to compare the results obtained with these two types of artificial lighting technologies.

### 1.1. Fluorescent Lamps

In 1901, the American Peter Cooper Hewitt (1861–1921) patented the first mercury vapor lamp technology (U.S. Patents No. 682,690/9) and the company General Electric developed the first practical and viable fluorescent lamp (U.S. Patent No. 2,259,040) that was first sold as a general market product in 1938.

These lamps consist of a watertight glass tube with a phosphor coating that contains a mixture of noble gases and mercury vapor in a low-pressure environment. The mercury content, which according to the ROHS and WEEE European Directives must be lower than 5.0 mg per lamp, makes its waste toxic and therefore its disposal and recycling must be properly managed [1].

The collision of electrons emitted by two tungsten filaments with the mercury atoms activates this gas and generates UV radiation which is projected towards the phosphor coating that absorbs and fluorinates them, emitting other radiations in the visible spectrum. The filaments consume a significant amount of electrical power generating heat. The energy transformation in a fluorescent lamp is presented in Table 1 [2].

Adapting the operation of these lamps to use Alternating Current (AC) from the electric consumption grid requires additional elements that manage both the start-up and the variation of the alternating behavior of the electrodes as anodes and cathodes. Thus, either one of these options is needed:

- Magnetic starter and ballast. These allow temperature increases of the lamp gas for its ignition, increasing the grid voltage with a reactance up to more than 1000 V_AC_. They can be accompanied by a capacitor to compensate the permanent inductive effect generated by the ballast coil. Dead cycles of the sinusoidal power signal generate a stroboscopic effect: in a 50 Hz network, the light oscillates 100 times per second, passing through its neutral position twice every 20 ms.
- Electronic ballast. This technology replaces the ballast coil of the magnetic equipment to generate the starting voltage peak with electronic circuits that transform the network signal to high frequency voltage and currents (20–50 kHz). This allows generating light with an emission flicker at this same rate, being less perceptible to the human eye. This increase in working frequency improves the energy efficiency of the tubes by reducing their consumption and lowering the degradation of their light flux. Thus, a fluorescent tube that consumes 18 W working with a magnetic ballast will only need approximately 16 W with a high-frequency electronic device.

From the point of view of energy efficiency, this auxiliary component introduces some additional consumption in the system due to the joule effect, which can be estimated as indicated in Table 2 [3].

Another aspect that affects the energy efficiency of the fluorescent lamp technology is the working temperature. In a generalized way, they are optimized for a nominal ambient temperature of 25 °C in which they achieve their optimum performance. With this configuration, in an environment at 45 °C, its efficiency in terms of the light output performance decreases by 20% and, conversely, at 10 °C it also drops by 25% [4].

### 1.2. Grolux Fluorescence Technology

Fluorescent lamps developed for people in domestic or working environments (offices and factories) focus most of their radiant energy within the visible spectrum band of the green and yellow colors. That is because it is in this segment where the human eye has the greatest sensitivity. However, the radiation emitted by these discharge lamps can be significantly modified using different combinations of phosphor films to suit other purposes.

**Figure 1:**
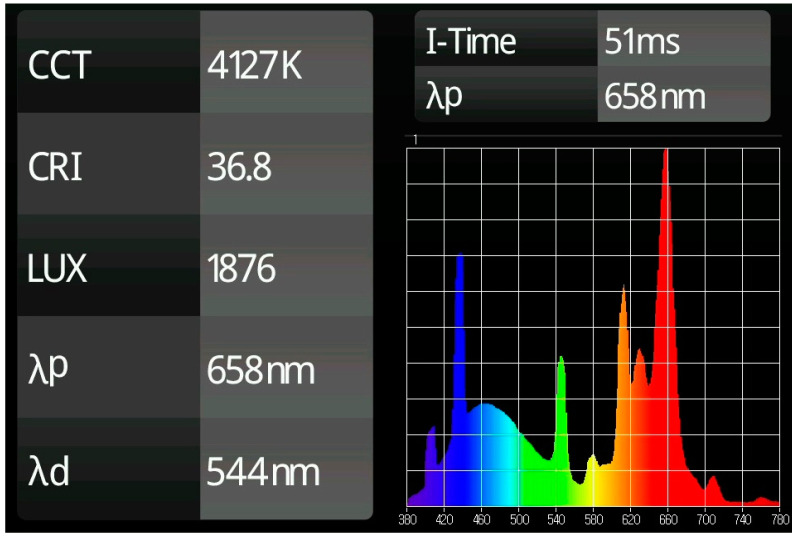
Spectral diagram and values of light characterization of the emission of a Grolux fluorescent tube.

Thus, the Sylvania Company began to commercialize a new version of fluorescent lamps in September 1961 under the trade name of Grolux (US SN 154,928) [5]. These fluorescent lamps emit most of their energy in two significant chromatic peaks: 435 nm (blue) and 660 nm (red). Since then, these Grolux lamps have been used as reference sources for in vitro plant tissue culture investigations and production. The visual sensation perceived by the human eye is slightly outside the range of the definition of the white color established by the International Lighting Committee (CIE of his name in French Commission Internationale de l’Éclairage) [6] and can be classified as an emission in the purple range. The Correlated Color Temperature (CCT) generated by this lamp could be assimilated to a neutral white light (4125 K) but with a very low Color Rendering Index (CRI) (37). This indicates that this technology has a very limited capacity to allow the perception of the complete set of colors available in the visible spectrum [380–780 nm] (see Figure 1).

Some of the most significant characteristics of these tubes according to their official technical data sheets at the time of development of this publication are presented in Table 3.

There is a variant known as Grolux WS (wide spectrum) that generates an emission spectrum of light in a more distributed way, adding more emission peaks, generating a more complete sweep of photons for a more natural color reproduction. Thus, while the Grolux light has a purple glow with a slight red appearance, the Grolux WS is white in appearance with a CCT of 3700 K. Nevertheless, this second technology has no relevant presence in the scientific literature concerning in vitro plant tissue culture procedures.

During the first years of the implementation of Grolux technology, studies such as those by Lammerts [7] or Helson [5] remarked how the lack of a methodological standardization of the tests led to studies on the same plant, using both Grolux and conventional fluorescence lamps to compare their performance and produced substantially different results. Helson [5] determined that different conditions of homogeneity of the light distribution or levels of radiance (i.e., number and power of the lamps used or separation between the light sources and the plants) marked these differences.

### 1.3. LED Lamps

At the end of the 20th century, experiments with LED lamps related to in vitro plant growth and morphogenetic processes began to be published and, in the first decade of the 21st century, in vitro culture growth chambers are being renewed, massively replacing fluorescent lamps with others based on LEDs. This renovation is done based on lower operation energy consumption and heat generation, and an advance radiant wavelength configuration capability that can be adapted specifically to optimize the biological processes of every analyzed species [8].

The LEDs, semiconductor electronic devices, are built by putting together two pieces of crystals, generally siliceous (Si), in which dopants have been added in a controlled way with an excess (N) or deficiency (P) of electrons in their valence layer. This union is capable of emitting light with a specific monochromatic wavelength defined by Plank’s law with respect to the energy difference between the valence levels of the electron of these dopants, when it is directly polarized by passing an electric current through its two sections.

The diodes were the first-born element of electronic technology for telecommunication purposes, with the first patent of these devices was registered between 1904 and 1906. However, as early as 1907, H.J. Round highlighted the observation of light emission phenomenon in these types of semiconductors. Nevertheless, it was not until the second half of the 1920s when Oleg Vladimirovich Losev [9] began to understand the reasons why light appeared in these devices. However, was is not until well into the 21st century that they have been standardized as devices with which to build robust and efficient lighting equipment.

Nowadays there are packages for both monochromatic lights, within the entire visible spectrum, as well as white light (continuous spectrum). This is thanks to the combination of high-energy blue spectrum LEDs (440–450 nm) and the use of photon conversion layers with phosphor using the same principle as the fluorescent light (White Light Phosphor Converted or WPC), to finally emit between 15–45% of green light. These last devices are very economical due to their massive production for general lighting equipment and take advantage of the beneficial effects of green light on plant physiology, photosynthesis and growth [10].

Beyond the spectrum of the light emitted, a wide variety of LED models or packages can be found on the market depending on different parameters and fields of application: luminous power, energy efficiency, light emission angles, thermal dissipation capacity, etc. From the point of view of power, they are usually classified into four families: High Brightness [HB] (range: 40–100 mW); Mid Power [MP] (range 0.2–0.5 W); High Power [HP] (range 1–3 W) and Chip on Board [COB] arrays (range: 3–100 W).

The circulation of current through the LED generates the aforementioned emission of photons but also causes electrical losses in the inner and outer connections and within the silicon crystal itself. This has a tendency to absorb part of the radiation generated, increasing its thermal energy and, consequently, its temperature. This increase in temperature, in the long term, progressively degrades the structure of the crystal, shortening its lifetime more markedly as the values that are reached become higher and, with immediate effect, causes the crystal to have a greater tendency to continue capturing photons. Therefore, a cooling system is necessary that prevents a possible catastrophic failure from excessive positive feedback [11].

HB LEDs are designed to provide pulsating variable visual information. They are manufactured from plastic and epoxy resin enclosures without any specific heat evacuation systems. This limits their useful life in the event of prolonged continuous lighting. In this mode of use, both the life as well as the efficiency and the amount of light emitted by the HB LEDs will be considerably reduced after approximately 10,000 h of use [12]. The rest of the LED families specifically contemplate heat dissipation systems. Nevertheless, they are developed intensively in the HP and COB models incorporating encapsulation with specific thermal dissipation channels. Their mission is to connect the substrate where the silicon crystal is placed with a metallic plane of the printed circuit board coupled to the heatsink of the luminaire, which are mostly metallic (aluminum) to favor the evacuation of heat to the ambient air. In this way, the average life established for these devices is an order of magnitude higher, up to 100,000 h. Therefore, in industrialized applications of continuous production, these LED models should be used to guarantee the durability and profitability of investments in lighting equipment, avoiding HB packages [13,14].

LEDs work naturally with an electric current in continuous mode (Direct Current or DC) and at very low voltage. Consequently, they also need auxiliary equipment to adapt the AC voltage of the electric grid to their required operation values. These power supplies, or drivers, are mostly AD-DC systems with Constant Current (CC) outputs that adapt to the quantity and power of the needs of the lamp’s emitters. They have an electrical efficiency of between 85% for low power lamps up to values close to or even higher than 95% [15]. Thus, the losses of the auxiliary equipment of LED lamps are lower than those needed by the discharge lamps. Moreover, the standard flicker of the light is minimum, as the output DC values of the drivers have very limited fluctuations.

The unreliability of these LED drivers was, at first, a limiting factor in the global life of lighting equipment, but technological developments in this field have already made it possible to guarantee lifetime expectations in the vicinity of 100,000 h [16], matching the values of the HP LED emitters.

Since 1997, when Miyashita et al. published the use of LED emitters to study the effect of red light on growth and morphology of *Solanum tuberosum* plants cultured photoautotrophically and photomixotrophically [17], interest in this type of lighting has been increasing and a large number of works have been published with different objectives and species. This information has been collected in reviews which, in addition to describing LED technology, have focused on aspects such as: effect of LED light source on in vitro plant growth and morphogenesis [18,19,20]; secondary metabolite [18,19,20]; production and related gene expression [8,20,21]; light and in vitro competence for photosynthesis, cell wall biosynthesis and in vitro propagation of woody species [8].

However, as [8] also pointed out, the primary issues regarding the lack of protocol reproducibility among laboratories are environmental factors, light being one of those. Thus, with the implementation of LEDs the same thing is occurring as in the 1960s with the Grolux fluorescent tubes, when Lammerts [7] and Helson [5] pointed out the need for a methodological standardization of lighting conditions.

## 2. Review Objectives

The main parameters and variables that must be taken into account in LED-based systems will be analyzed due to the wide range of possibilities they offer, contrary to what happens with Grolux fluorescent tubes with which the capability to configure factors is very scarce. These parameters and variables will therefore require a description and quantification under experimental conditions in order to homogenize and standardize the experiments and, thus, to obtain reproducible and comparable results and conclusions.

This paper reviews recently published studies that include the use of LEDs on in vitro plant tissue culture, to know, among the most relevant parameters in terms of lighting conditions, which are described in the experimental systems of the reviewed papers and what methods are used to measure or characterize these parameters. Next, the advantages and drawbacks of these alternatives used are highlighted.

In order to use LEDs as a standard lighting source in in vitro growth chambers for the maintenance of previous in vitro culture procedures without affecting their behavior, it is desirable to be able to reproduce the lighting conditions universally used in in vitro culture using LED technology. For this objective, it will be necessary to (i) configure the LED lamps so that they emit in the same spectrum as the reference Grolux fluorescent tubes, (ii) to configure desired light irradiance values and (iii) achieve large uniformity of radiation conditions on the shelves of the culture cabins.

This review is contrasted with the results obtained in an experimental system in which artificial lighting is supplied in cabins with, in some cases, Grolux fluorescent lamps (Sylvania, OH, USA) and, in others, with a LED lighting system calibrated to offer a spectral emission, irradiation level and uniformity similar to that generated by previous discharge lamps.

A greater perspective to understand the effect of LED lighting on in vitro plant growth and morphogenesis is intended which may allow the characterization of the response of each species to establish optimum intensity, distribution photometry and wavelength conditions for in vitro plant tissue culture. This will determine the most suitable LED lighting equipment and configuration for each species and biological processes.

## 3. Characterization of LED Luminaires for Use in Plant Tissue Culture

The large number of works published in recent years using LED technology in plant tissue culture (Table 4) show that interest in this type of light source continues to grow. This is due to the fact that the use of LEDs has opened a huge field of possibilities where light has gone from being a determining factor for photomorphogenesis, but with little manipulation capacity, to being a component that can be configured very specifically, allowing the control and manipulation of in vitro plant growth and morphogenesis.

However, while the evaluation parameters of the results of the studies and investigations analyzed are, in general, very similar or equivalent, the description of the experiments carried out reveals a clear heterogeneity of configurations and approaches from the point of view of the lighting installations. This coincides with what is indicated by Batista et al. [21], who pointed out that the primary issues regarding the lack of a reproducibility protocol among laboratories are environmental factors and that light (in quantity and, particularly, in quality) is one of those main factors.

The lighting aspects involved in the photonic processes of enhancement of biological processes are multiple and their adequate standardization or, at least, their precise characterization in experiments is essential to allow a comparison analysis between different results (as they are measured under equivalent conditions).

The most relevant design fields to take into account in this type of installation are described and analyzed in the next chapter. While in systems based on Grolux fluorescent tubes there is very little capacity to configure working parameters, apart from the mere selection of the number of lamps, their electrical power and the separation distance from the plants to achieve the radiation level desired, there are a wider range of possibilities with LED lamps.

We propose a list of possible parameters and variables to be characterized in LED installations due to their influence on the development of in vitro plant tissue cultures. Later, the information found in the description given in a comprehensive review of scientific publication will be compared with this list. The advantages and disadvantages of the different alternatives used for the descriptions of the lighting equipment and how it was configured will be highlighted. We consider that it is essential to establish these criteria to homogenize and standardize the experiments that are detailed, indicating in the description of the installation of LED luminaires, at least, the following elements using the units of measurement that facilitate more precise comparisons.

Of all these variables, two are fundamental: (A) the precise clarification of the wavelengths of the radiations that are emitted (considering three different cases of lamps with: one type of monochromatic emitter, with a combination of these and with a set of diodes that generates a continuous spectrum) and (B) the values of irradiance that are projected on the plants as a measure of the amount of energy that is being injected. However, related to the two previous variables, the guarantee of (C), the uniformity of the lighting conditions achieved on the shelves in the growth chambers is also needed. Due to the discrete nature of the encapsulated LEDs and their directional emission of light, this requires detailed planning to guarantee adequate values.

### 3.1. Characterization of the Emission Spectrum

This information can be obtained from the LED or lamp manufacturer or, more exactly, measured specifically with a spectrometer. It should be specified precisely and specifically:

- In monochromatic emissions, the wavelength value of the emitters used must be indicated without simplifications of those group values, i.e., defining a radiation spectrum not by its peak wavelength but by the generic name of its color as red (that globally identifies all the range between 600–700 nm). Based on the widths of the radiation peaks offered by commercial LED packages and the variability found by the precision of the manufacturing mechanisms of these solid-state emitters, we consider that a sufficient value precision is to contemplate differential steps of 10 nm (e.g., 650, 660, 670 nm).
- In emissions that combine several monochromatic light sources, each color should be described as in the previous case. Moreover, the ratio of each type of radiation should not be assessed solely on the basis of the relative number of LEDs of each of the colors. Since the efficiency, power, thermal behavior and degradation curve of each type of LED is not homogeneous, this cannot be considered a comparable reference. Thus, the relationship between the light sources should be characterized by the energy radiated in each frequency. This information is offered by the graphical representations of spectra of the light that can be obtained with a spectrometer or can be obtained measuring with the PAR meter the radiance of each color independently.
- In the case of lamps with continuous frequency range emissions within the radiant spectrum, i.e., white light, it would be necessary either to establish their commercial identification, if it exists, or to show, preferably, the radiation emission diagram of the light source used obtained with a spectrometer, which gives the exact information proportions of each emitted radiation. However, since it is very difficult to accurately compare two continuous data curves from printed images, the specific values of the Correlated Color Temperature (CCT) and the Color Rendering Index (CRI) are also significant characterization elements of this type of emissions.

The CCT or the color temperature of a light source is the temperature that makes an ideal black body that radiates light of a color comparable to that of a light source. From a practical point of view, in LED lamps this variable gives a general idea of the proportion of radiation in the blue spectrum (450 nm) and the green/yellow spectrum: more blue than green in 5000 K and higher; equivalent peaks of radiation in 4000 K and more relevance for green emissions in 3000 K and lower.

The CRI measures the reproducibility of the colors of a light source in comparison with the incandescent bulb that is used as a reference of a continuous spectrum of emission from a black body. Because the red color segment has little influence on this CCT measurement, this variable, for white LED light, indicates that at values above 80 the red component of the spectrum reaches top values on commercial white LED light, while, in 60–70 CRI emissions, the number of photons within the red color range are low.

#### Review Results. Identification of the Spectral Values of the Light Sources Used

The three different groups of lamps found are analyzed separately: fluorescent light, LED white light, LED monochromatic light and a combination of them.

- Fluorescent light: A major conclusion that can be obtained is that the characterization of LED lamps is carried out in greater depth than that of fluorescent lamps that are used as a control element in many of the experiments studied. From 62 papers that use fluorescent lamps as the control lighting system, 30 of them (48.4%) do not give any specific description or details about the type of lamp used; another 15 (24.2%) only give a generic description of the group of the color that is emitted (cold, neutral or warm) but without any quantitative characterization value; and only the last 17 descriptions of works (27.4%) either present the graph of the radiation spectrum of the lamps (three papers) or quantitatively determine the nature of the light source used (CTT for white light or the description of the specific model of lamp with its identification of the spectrum through the manufacturer dataset and, thus, of the radiant spectrum that is emitted on the plants) (14 papers).
- In this assessment we highlight an increasing trend in the presentation of details of the characterization of lighting equipment in the works that have been published more recently compared to older ones, due to the use of spectrometers in in vitro plant tissue culture laboratories, among other reasons.
- White LED light: The main deficiency detected in the characterization of these type of LED lamps refers again to the sources of white light, since the weight of each of the wavelengths in this continuous spectrum emission is not defined in a clear way. Describing only extreme wavelength values does not allow us to establish whether the main emission weight is in blue light (440–450 nm) or in the yellow-green zone. In this case, a minimum description would require an indication of the color range of the white light (cold, neutral or warm). On the other hand, the most precise description is to provide the full spectral graph of the emitted light obtained with a spectrophotometer. An intermediate solution, which offers sufficient values for comparison purposes due to the standardization that is being found in these products, is to offer the CCT and CRI values of the light source. From 57 studies analyzed that claim to use LED white light, four offer the graph of their radiation diagram; nine indicate the CCT value (with values from 2700 K to 7000 K, the most common numbers being 3000 K, 4200 K and 6500 K); eight identify only the range of CCT with one of this three options: cold, neutral and warm (the first and the last values being the most identified) and, finally, 36 indicate simply that white LED light has been used without any extended specification.
- Monochromatic LED light: In these cases, each light source can be clearly identified by the wavelength of its emission. It is relevant at this point to note how the generalized denomination of a color does not correspond to a single spectrum but to a set of frequencies that offers slight visual differences. In total, 52 studies out of 92 clearly identify the peak wavelength values, while the remaining 40 were limited to identifying only a generic group definition (red, blue, green, etc.). As described above, it is noteworthy how each plant species reaches a point of maximum sensitivity at different frequencies, so a precise study would require using not only an emission value in each basic group of colors, but also in making a sweep in several discrete frequencies. In the cases in which this more specific discrimination is done, there are 19 studies that deal with differences in the red color, including the study of the effect of far red or infrared lights (>700 nm) and seven studies include, at least, two different frequencies inside the range of the blue color (425–475 nm). Five papers introduce emissions with UV values (<400 nm) and another three use the violet/purple region of frequencies (400–425 nm).

### 3.2. Amount of Radiated Energy Incident on Plants

This is a major technical value presented in most of the descriptions available in the literature but is treated in different ways.

Most common technical variables related to lighting equipment and installations are defined based on the behavior of the human eye. The luminous flux—measured in lumens—is the total amount of energy emitted by a lamp, and a technical value given by their manufacturers to identify the products. However, the lumen is a unit of measurement that expresses how bright a certain light appears to the human eye and favors the yellow/green/orange spectrum because of the human eye’s spectral sensitivity curve. The counterpart option is the Photosynthetic Photon Flux (PPF). That unit measures the quantity of photons that a light fixture emits per second within the segment of the radiation spectrum that affects the photobiological processes of the plants: the Photosynthetically Active Radiation (PAR) region. This is defined between 400 nm and 700 nm. The unit of the PPF is the µmol s^−1^. However, neither of these two variables are adequate to characterize an experiment, as they give the total energy emission, and it is not possible to accurately assess how much of that radiation is directed towards the plants and how much is lost in the surrounding environment.

Thus, in order to assess an experiment correctly, it is necessary to define the measurement of the amount of energy incident in the plants such as the irradiance, or the radiant flux received by a surface per unit area. This effect, again, can be represented using different units. The International System (IS) unit is the W⋅m^−2^ but its usefulness is more suitable for energy balance studies, as it considers the full spectrum of radiations rather than identifying the adequacy and quantification of the number of photons within the PAR region. Moreover, irradiance meters are both costly and limited in the ability to measure low irradiance values. With a lower cost and higher sensitivity in low light conditions, basic light meters measure the luminous flux per unit area (illuminance) utilizing the units of lumens per meter squared or lux. As before, this does not accurately reflect the number of photons that reach the plants with different light sources because it is adapted to the perception of incident light by the human eye (International Electrotechnical Commission EV Ref: 845-21-060), i.e., it does not quantify the radiations in the range of the green-yellow spectrum in the same way as in the blue or red segments.

Considering the above, the most appropriate measurement variable, when working with plants, is the Photosynthetic Photon Flux Density (PPFD), with µmol m^−2^ s^−1^ as its unit. This is the amount of power of the electromagnetic radiation received on a surface measured as the number of photons in the PAR region of the spectrum received in a square meter per second [118]. This variable can be measured with affordable PAR sensors which are manufactured to offer a response in which each photon is equally absorbed considering that photons of shorter wavelength (higher frequency) have more energy than photons of longer wavelength.

For standardization reasons and to facilitate processes, the PPFD values should be measured on the flat surface where the culture vessels are placed.

#### Review Results. Characterization of the Amount of Light Irradiated

With regard to the irradiance of light, this variable is intended to be quantified in most of the descriptions reviewed. Overall, 85 of the works analyzed (87.6%) made a description of quantitative experiments against another 12 papers (12.4%) that did not specify this information in any form. The most widely used variable is the PPDF (µmol m^−2^ s^−1^) that is an option presented in 78 works, compared to two works that indicated the characteristic radiation of the light source (W m^−2^) and another six of them characterized at least one experiment using illuminance (lux) values.

The values of irradiance used in the different experiments are variable. Standard values in this field are 45–50 µmol m^−2^ s^−1^, and a significant number of the studies analyzed use these values. However, others use different quantities that are found mostly within the range among 25–85 µmol m^−2^ s^−1^. Dispersions are found in those works that either: (A) include analyzing the consequences of using different radiation intensities, (B) use different values justified in previous studies that indicate that these other quantities optimize a specific biological process on a species of plant or, finally, (C) present different values of µmol m^−2^ s^−1^ for each different light source in their experiments.

Regarding this last case, it may be significant to indicate that the discrete power values of the fluorescent tubes and their unique photometric emission diagram facilitates obtaining the previous standard values based on previous experiences, choosing the height and number of tubes depending on the surface of the cabins. However, the large heterogeneity of LED lamps makes it difficult to foresee the value to be obtained correctly and dispersions from these expected values can be easily obtained if a previous analysis bases on simulations or similar is not done. Including a dimming system allows the precise configuration of the installation as desired but if that option is not available, as in basic LED lamps, the only method available to adjust the intensity sent to the plants requires the modification of the geometry of the cabins.

### 3.3. Uniformity of the Lighting Conditions Achieved on the Shelves in the Culture Room

**Figure 2:**
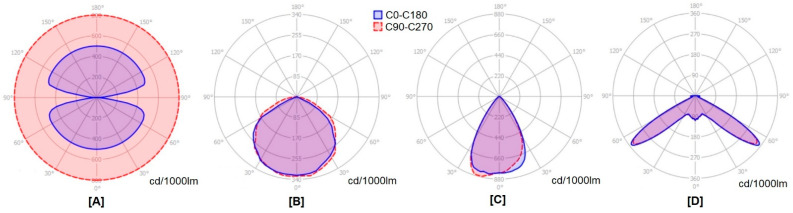
Common photometric diagrams found in led lamps for in vitro culture. (**A**) Fluorescent tube lamp (**B**) 120° hemispheric distribution (regular pattern of most HP LEDs without external lenses) (**C**) 60° concentrated distribution (regular pattern of many HB LED without external lenses). (**D**) Homogenized 120° distribution. Optimal lens design for chambers (i.e., PMMA C17720 EMERALD lens by Ledil).

One of the characteristics that makes fluorescent tubes, both cool/warm white or Grolux light, a very suitable type of lamp for supplying light in in vitro culture rooms is the high uniformity of the light that they emit and project over the plants. The continuous, homogeneous and omnidirectional nature of the light emitted by fluorescents (See Figure 2A) makes obtaining high values of uniformity much easier, even without a specific study of this parameter. To reproduce lighting conditions similar to those provided by fluorescent tubes using LED technology, it will therefore be necessary to achieve ranges of the same level of uniformity of radiation conditions on the shelves of the culture room.

Contrary to what happens with fluorescent lamps that emit continuously and in all directions of space, LEDs are discrete light sources, integrated into non-continuous matrixes that are distributed over the entire surface of their lamps. In these matrixes, emitters of different spectra are usually combined. Moreover, the light that they emit is directional and has several possible configurations depending on the type of LED used and if any secondary optic component is integrated in the lamp (see Figure 2B–D). This has the consequence that the light reflected from the walls of the chambers has a much lower relevance than with fluorescent tubes, since the light is projected in a more concentrated way towards the work area. This is an obvious advantage from the point of view of energy efficiency, but it requires that the geometric configuration of the specific lamps being used and their position in space must be analyzed in order to guarantee that the same amount of light (both in intensity and in wavelength of radiation) reaches the entire vessel-laying surface. It is necessary to measure and guarantee a high surface homogeneity of the radiation emitted in its two mentioned variables to prevent the projections generated in the in vitro cultures placed in different locations within the working zones being significantly different.

Overall, Figure 2 compares the omnidirectional emission of the fluorescent tubes (Figure 2A) (radiations are not present only in the direction of its electrical connection sockets as can be observed in the transverse plane represented by the blue curve) compared to the natural directional nature of the LEDs (Figure 2B). The latter allow a simple and highly efficient adjustment of their emission curves by means of secondary lenses, as in Figure 2C,D.

Fluorescent lamps emit a lot of light outside the reach of plants, and it is necessary to play with the reflection of the working cell (walls and ceilings) or with specific reflectors to redirect the radiations. This reflected light system is not very energy efficient, but it allows homogeneously illuminated environments to be obtained in a natural way. However, the directionality of LEDs greatly optimizes energy efficiency but requires a configuration study of each case to ensure that there is uniform incident radiation throughout the work surface for all emitted wavelengths.

The two standardized systems to measure the uniformity of light emission are the average uniformity and the extreme uniformity. The first is defined as the quotient between the minimum value of the incident radiation, measured on our useful working area divided by the average value obtained from all the discrete value calculations used to characterize this surface (see Equation (1)). On the other hand, extreme uniformity is the quotient between the minimum value and the maximum measured value. These homogeneity values should be published for each of the dominant emission frequencies used in the experimentation spaces. To calculate these uniformity values of average and minimum radiance, a regular 2D matrix of test points must be marked over the complete working plane, with a step distance smaller than the diameter of two sample vessels (i.e., 15–20 cm) and equal in both Cartesians directions.

(1) $$\mathrm{Umean}=\frac{\min{\left({\mathrm{PPFD}}_{i,j}\right)}_{i=1:n;j=1:m}}{\sum_{i,j}^{n,m}{\mathrm{PPFD}}_{i,j}}$$

With ‘n’ being the number of discrete test points in the ‘X’ axe and ‘m’ the number of discrete test points in the ‘Y’ axe.

The radiance of each test point configured should be collected using a PAR meter for every type of emitter available. Only one value is needed for monochromatic of single-type continuous spectrum LEDs, but for LED lamps with several different emitters where each spectral emission can be controlled independently, this operation must be repeated for every case where a single color can be turned on independently. As an alternative, for homogeneity measure purposes, a lux meter can also be used as sensor without loss of accuracy because the relative condition of the variable only requires a homogeneous system of measure.

Once data are collected, each minimum irradiance is identified as the numerator of the uniformity ratio and the denominator is the average radiance that is obtained adding all the values and dividing the result by the number of points measured. Our recommendation is to consider that good values of average uniformity are ratios above 0.70 and they become optimal above 0.80.

#### 3.3.1. Review Results. Characterization of the Uniformity of Light Radiated over the Plants

None of the works analyzed in the review presented any quantitative measure or consideration of homogeneity of either intensity or wavelength of radiance values, rather they offered statements of intent of subjective visual appreciations.

#### 3.3.2. Experimental Verification

**Figure 3:**
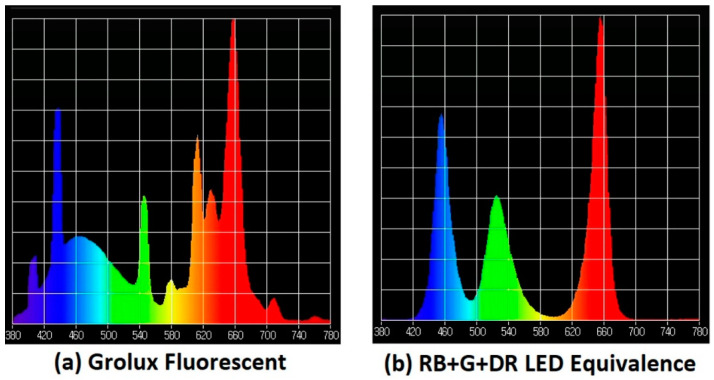
Comparison of the light spectra of (**a**) Grolux fluorescent and (**b**) R + G + B LED equivalence.

Figure 3 presents the comparison between the Grolux fluorescent tube spectra and the LED luminaire designed by the authors to offer an equivalent functionality. This equivalence is based on the proportional reproduction of the radiation of three monochromatic wavelengths (Blue (440–450 nm), Green (540–530 nm) and Red (660 nm)). The number of emitters and their geometric placement was established to homogenize accurately the lighting values achieved throughout the working area with the plants.

**Figure 4:**
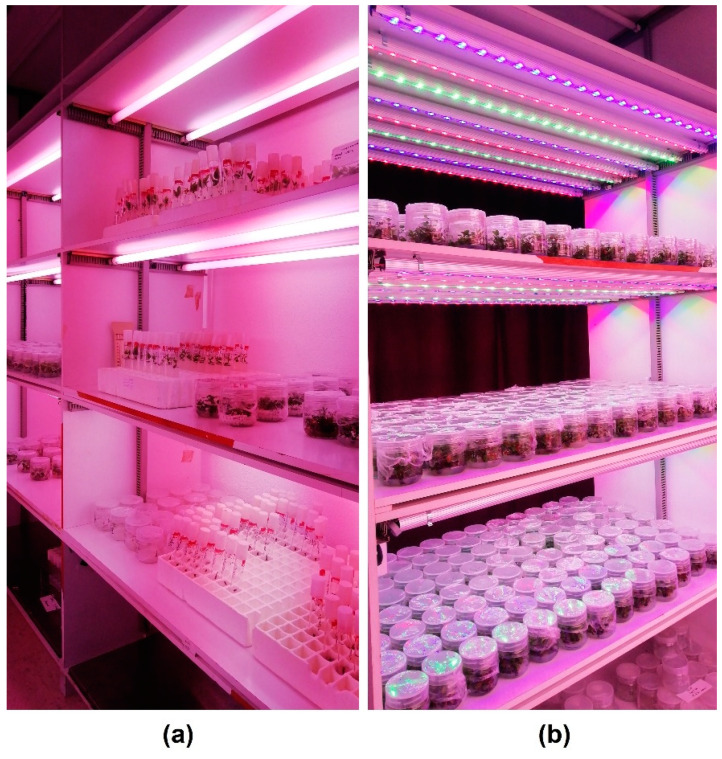
Test cabins with (**a**) Grolux fluorescent lamps and (**b**) LED Grolux spectrum equivalence.

As reference, a model cabin measuring 130 cm (length) × 59 cm (width) × 44 cm (height) has been designed. In this working cell, six linear LED lamps, 115 mm long, have been attached to the upper wall. Each tube is built using only one type of light source in the color spectrum and has a mechanical structure that is formed by an extruded and anodized aluminum heatsink (to improve the heat evacuation capacity by natural convection) with a diameter of 13 mm. Each of these lamps contains 24 HP monochromatic LEDs from the manufacturer CREE with a separation distance of 4.5 mm without secondary optics. There is therefore a hemispherical projection of 120° (See Figure 4). Each tube is placed in the cell 8 cm apart from each other and centered with respect to the cabin and combined alternately with a pattern: R, G, B, R, G and B.

An SQ-120 quantum sensor by APOGEE was used to measure the uniformity that is achieved throughout the useful surface of the cell. This measurement equipment was chosen based on its low thickness deployable sensor that allows the least possible interference in the measurements by any shadow. A measuring mesh matrix of 12 × 5 (60) equidistant points was set starting from four fixed positionings located on the corners of the surface located 2 cm apart from the two edge lines of the cabin.

The mean uniformity of each of the three wavelengths that make up the equivalence of Grolux tubes were independently measured. In all cases, similar average uniformities were obtained around 0.72. The minimum readings were always given at one of the four points associated with a corner. In the case of excluding the measurements of these points, the average rose to 0.79.

**Figure 5:**
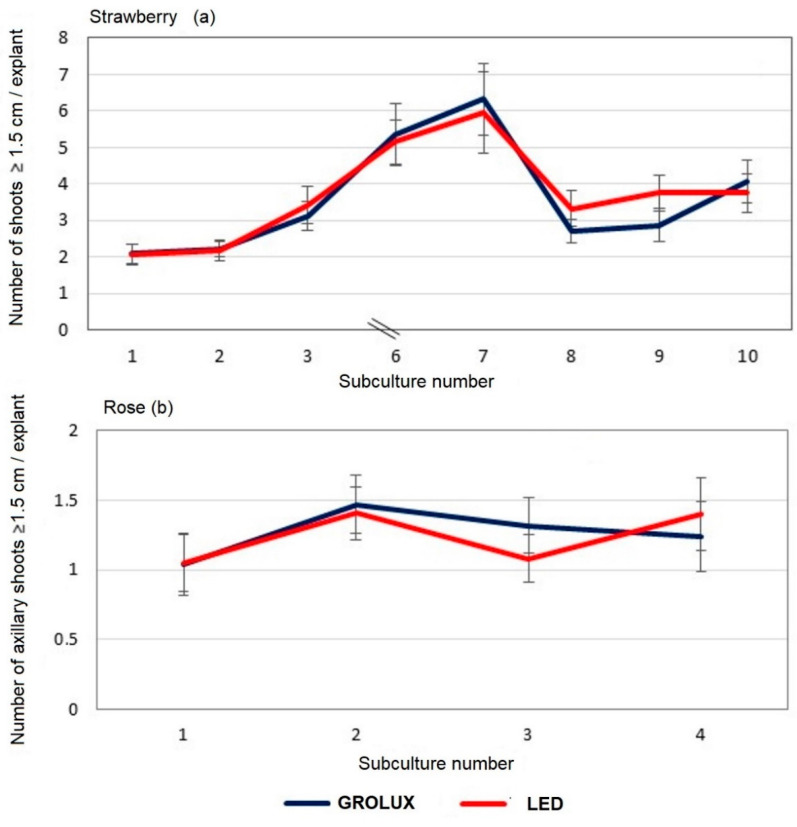
Effect of the light spectra on in vitro proliferation of (**a**) strawberry and (**b**) rose with Grolux fluorescent (blue line) and R + G + B LED equivalence (red line).

With the lighting conditions of spectral composition and homogeneity previously described, and using strawberry and rose as model plants, practically identical results were obtained during successive subcultures in the proliferation of these two species when using Grolux and RGB LED tubes with the equivalent configuration [119]. See Figure 5.

### 3.4. Temperature

Fujiwara and Kozai [120], reviewing the physical microenvironment and its effects, pointed out that the temperature of the air within the culture vessels can be considered almost the same as that outside the vessels during almost all the dark period and that, during the photoperiod with cool white fluorescent lamps, the air temperature differences between the inside and outside were less than 0.5 °C with an irradiance of 60 µmol m^−2^ s^−1^. With this data as reference, no special attention needs to be paid to the air temperature in culture vessels in conventional plant tissue culture. However, these temperature differences increase with the raise of the irradiance values, as Tani et al. [121] pointed out, being maximum at the surface of the culture medium.

Incandescent and discharge lamps have electrodes and gases (in the second case) that are activated at high temperatures for their operation, which means that they emit a significant amount of radiant heat emission (infrared) in the same direction in which they project light. In addition, the electrical losses in the auxiliary work systems and in the connections of these lamps generate an additional amount of heat that is transmitted in the air through non-forced convection, directly affecting the culture vessels. In contrast, in LED lighting systems there are no components that reach high temperatures, eliminating the direct transmission of heat by radiation.

#### 3.4.1. Review Results. Effect of Light Source, Discharge vs. LED, on the Temperature in Plant Tissue Culture

Considering the works covered in the review process, there are no data presented in the bibliography about the effect that their light sources have on temperature in plant tissue culture. Thus, it is not possible to compare the differences produced on the temperature at which cultures are found using fluorescent or LED lighting.

#### 3.4.2. Experimental Verification

**Figure 6:**
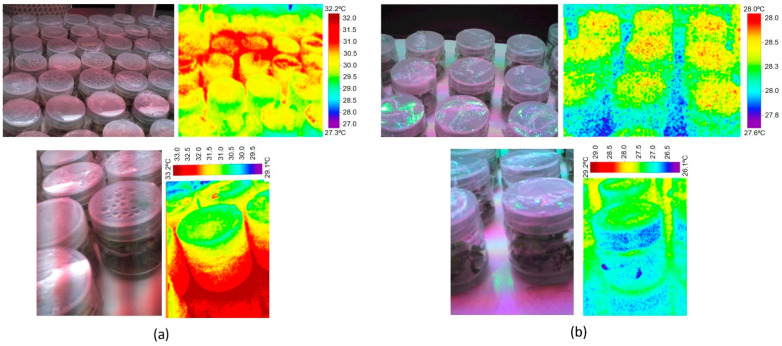
Thermographic images of the temperature outside the vessels under (**a**) Grolux fluorescent lighting and (**b**) LED lighting. (Thermographic camera model 875. Manufacturer Testo).

Measuring the temperatures inside and outside the culture vessels in the experimental conditions previously described, it is possible to observe that under Grolux lighting vessels reached a temperature of 33.2 °C and condensation frequently appeared in the culture flasks, especially in those items located above or below the line of the fluorescent tube (Figure 6a). On the contrary, under LED lighting no condensation appeared in any jar and their temperatures did not exceed the 28.8 °C (Figure 6b).

The elevated temperature found on the shelves, generated by the heat transmitted conductively though the material of the racks, made the temperature inside the vessels rise more than the outside ambient values. This produces an increase in the relative humidity in the plant ambient and a decrease in the inter-gaseous change, a phenomenon that Pasqualetto [122] called the ‘greenhouse effect’, which plays an important role in the appearance of hyperhydric plants.

**Figure 7:**
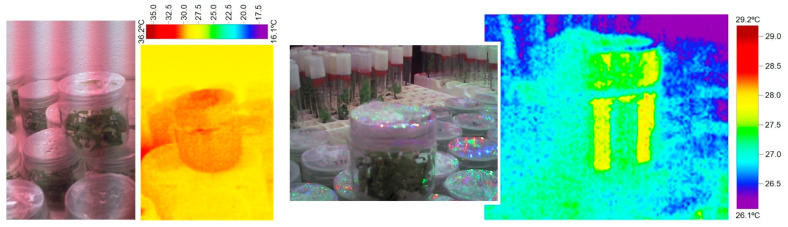
Thermographic images of the temperature in the vessels under (**a**) Grolux fluorescent lighting and (**b**) LED lighting. (Thermographic camera model 875. Manufacturer Testo).

Observing thermographic images, it can be seen that the temperature ranges inside the vessels oscillate between 27.5–35.0 °C while using Grolux lighting (Figure 7a) and between 27.0–28.0 °C (Figure 7b) when LED lighting is used. Therefore, depending on the area of the container, there would be differences of up to 7.5 °C with Grolux compared to only 1 °C with LEDs. Therefore, although temperature is not a parameter intrinsically linked to the light source, it will have a differential influence on the temperature reached inside and outside the culture vessels and on the temperature gradient created inside these containers.

## 4. Future Trends: Pulsed Light

In addition to these previous parameters of which information is found to a greater or lesser extent in the research works analyzed, it would be significant to detail also the constant or pulsating light flux emission identification. Whereas fluorescents always emit with a certain fluctuation due to the dependence of the sinusoidal signal of the power supply network (50/60 Hz, depending on the country), LED lamps work on constant current condition with no effect (theoretically) of oscillations. The drivers or power supply systems of LEDs that convert the electric power from AC to DC can filter the sinusoidal fluctuations of the standard power grid to achieve light emissions with an almost zero level of oscillation, at best (flicker < 1%), or higher (>10%) in lower quality products. However, the electrical nature of LED and its fast-switching capability allows the generation of variable active width control signals with supplementary regulation devices that can be used to generate different pulsed emission conditions. Pulse Width Modulation (PWM) regulators that create variable square signals commuting the input of electric power of the light sources are a common technology used to dim, in a controlled way, the intensity of light emission of LED lamps.

Various past [123] and recent works [124] have stated that this can be a variable that significantly influences different plant processes such as biological absorption. Thus, this can be considered a variable of interest in in vitro plant tissue culture, although there are still very few studies in this field, such as [125], which leaves open a line of investigation. In this case, it is important to characterize the shape (square, sinusoidal, etc.), amplitude, width active percentage and frequency of the light pulses configured to facilitate comparison of different experiments. These values are introduced as required inputs of a lighting installation control system to obtain one specific output setting which can be measured and verified using a spectrometer with flicker measurement capability.

## 5. Concluding Remarks

A large number of works are being published both in a general sense and, more particularly, in the area of in vitro plant tissue culture, in which LED lighting is used and its effect on growth and morphogenesis is evaluated. The nature of technology is significantly different compared to that the fluorescence, which has been most commonly used in the implementation of artificial lighting in in vitro culture rooms. The LED flexibility offers significant advantages (configuration, control capability, etc.) but also presents the problem of establishing how to guarantee that the results of published works are extrapolated, reproducible and comparable, not only for LEDs but also for fluorescence.

This review has focused on the need to standardize lighting conditions and their description. To achieve this objective, and having considered the state-of-the-art technologies involved, the characteristics and technical variables that must be detailed have been established so as to propose a methodological standardization. In this sense, it can be considered that there are three control parameters necessary to achieve this objective: incident radiant intensity in the crops, detailed light emission spectrum and uniformity of incidence of these radiations on the plants.

The description and analysis of LED or mixed in vitro lighting installation presented in 97 scientific publications since 2018 to the present day were reviewed and they were assessed with regard to the proposed variables and methodology. It is noteworthy that in general there is still a great need to improve the amount of detail in these descriptions; however, that said, the depth and precision of the descriptions found in many recent works compared to the oldest works analyzed show a positive trend in this sense.

Together with this bibliographic study, an experimental contribution has been made in which two lighting installations (Grolux fluorescence and LED) have been designed and configured to offer equivalent lighting performance considering the generated irradiance obtained in the shelves, emission spectrum—built based on the three main peaks of wavelength generated—and uniformity of both previous parameters. This experimental setup has made it possible to verify that the results obtained in the in vitro proliferation of strawberry and rose, as model plants, are practically identical with LED lamps configured and installed to match the emission of the Grolux fluorescents.

Finally, another noteworthy result of the study is how, although temperature is not a parameter intrinsically linked to the light sources, the lamps chosen—LED or fluorescence—will have a differential influence on the temperature reached inside and outside the culture vessels and on the gradients of temperature created. The increment on the environmental and microenvironmental temperatures generated is smaller when using LED technology.

## Figures and Tables

**Table 1:** Average distribution of the transformation of electrical energy in fluorescent tubes.

Energy Conversion	Visible Light	Ultra Violet (UV)	Infra-Red (IR)	Heat
Fluorescent lamps	23%	1%	30%	46%

**Table 2:** Range of energy losses depending on the type of auxiliary ballast of fluorescent lamps [3].

Conventional Magnetic Ballast	High Efficiency Magnetic Ballast	Electronic Ballast
20–25%	14–16%	8–11%

**Table 3:** Range of energy losses depending on the type of auxiliary ballast of fluorescent lamps.

Technical Parameter	Quantity
Mercury content of the lamp	2.80 mg
Control gear required	Electronic Ballast (+8–10% Power)
Average life (Nominal)	14,000 h
Operating temperature range	−15–40 °C (Maximum luminous flux @ 25 °C)
Light color	Grolux (Chromaticity Not adjustable)

**Table 4:** Experimental LED lighting conditions on in vitro plant tissue culture. (A): Lamps, spectrum. (B): Radiation, measurement equipment, laying distance.

Species	Experimental Lighting Conditions		Parameters Studied	Remarks	References
	(A)	(B)			
*Aeollanthus suaveolens*	LEDs: W, G, Y, B, R, R:B (1:2.5, 2.5:1; 1:1) FL: CW (C) Graph spectrum but not given definition values or charactersitics	PPFD LED (μmol m^−2^ s^−1^): W [20, 57, 78, 102 and 139]; Monocromatic LED: undefined PPFD FL (μmol m^−2^ s^−1^): 42 SPECTRA PEN Z850 spectrometer; Qubit Systems QSO-S Procheck + Sensor-PAR Photon Flux device; Decagon Devices-Pullman.	Shoot and root no. and length; leaf no.; shoot, root and leaf DW; leaf area; total DW	Growth was optimal with a PPFD of 139 μmol m^−2^ s^−1^, CNOC and CHL content, increased at 20 μmol m^−2^ s^−1^. W LEDs and FL were similar for DW and growth, but R, B and Y LED inhibited the last	[22]
*Stevia rebaudiana Bertoni*	LED (nm): W (420) -undefined-, R (660), B (460) IP65 model, SMD 5050 RBG (Techno Lite^®^). FL: (4000–7000 nm) (C) -Typing error and undefined-	Illuminance (klux): CW FL 1, 2, 3 and 4 PPFD LED (μmol m^−2^ s^−1^): 40–50	Shoot induction %, no. and length; leaf no.; FW; DW; callus formation; CNOC and CHL content	High-quality plants can be grown in vitro under B, R and W LEDs for mass propagation and genetic improvement	[23]
*Clerodedum indicum* and *Acanthus ebracteats*	LED (nm): W (400–700), B (425–500) and R (600–700) -undefined-	Irradiation: 61.5 W·m^2^ 30 cm between light sources and plants	Verbascoside level	Methyjasmonate and B LEDs enhanced verbascoside level	[24]
*Urtica dioica* L.	LED: B, R, W and B:R (1:1, 2.5:1, 1:2.5) -undefined- FL: CW (C) -undefined-	PPFD LED (μmol m^−2^ s^−1^): 26, 51, 69, 94 and 130 PPFD FL (μmol m^–2^ s^–1^): 42 Portable spectrometer SPECTRA PEN Z850; Qubit Systems-Kingston. QSO-S Pro Check +Par Photon Flux Sensor; Decagon Devices; Pullman	Plant and root length, DW, leaf area, photosynthetic pigments, phenolics, favonoids and antioxidant activity	A PPFD of 94 μmol m^−2^ s^−1^ stimulated growth and DW. The antioxidant activity was directly proportional to the increase in light intensity	[25]
*Brosimum gaudichaudii*	LED (nm): W (400–700), B (400–490), R (645–700) and R:B (1:1, 1:3, 3:1) -undefined-	PPFD (μmol m^−2^ s^−1^): 100 ± 5 PAR sensor, model APG-SQ-316; Apogee	Shoot length, leaf no. and area, stem and leaf dry matter, total dry matter	R induced elongation. B and R:B enhanced the content of furanocoumarin, but B:R decreased the growth	[26]
*Zingiber officinale Theilade*	LED (nm): W (400−700), B (460), G (530), R (660), fR (720) and R:B (1:1) (400 and 660) LED Tubes, OSRAM Opto Semiconductors	PPFD (μmol m^−2^ s^−1^): W 15.3, fR 20.8, B 15.8, G 17.0, R 29.3, R:B 44.8 30 cm between light and plants	No. of Microsprouts, shoots, roots and leaves; shoot length; porphyrin, CHL and CNOC content	R LED improved rooting and shoot length, P stimulated micropropagation. G, R and P enhanced microshoot growth considerably, but fR and B, stimulated it too	[27]
*Rosa x hybrida cv. Sena*	CW FL cold bulbs 40 W & W LED bulbs 10W -undefined-		Shoot regeneration %, flowering induction %, abnormal flowers %	W LEDs (10W) reduced the % abnormal flowers in vitro, similar to W FLs (40 W)	[28]
*Capsicum frutescens*	FL (Luz do Dia Especial) (40 W, Osram) -undefined- LEDs: W, R, B and R:B [6:4] (TEC LAMP^®^) -undefined-	PPFD (μmol m^−2^ s^−1^): 72	Germination %; length; nodes no., no. of green and senescent leaves; FW, DW, CNOC, CHL	Porous membranes, R and B:R improved growth and development	[29]
*Scrophularia kakudensis*	LED (nm): R (621 and 710), B (450 and 475) (4 lamps PSLED1203-50A, Force Lighting) FL: CW 40 W, Philips -undefined-	PPFD (µmol m^−2^ s^−1^): 50 30 cm between light sources and plants	Growth, stomatal ultrastructure, phenols and flavonoids, activities of antioxidant enzymes, and protein expression	B or R LEDs improved the micropropagation. R and B elicited the synthesis of secondary metabolites	[30]
*Droseraceae*	LED: CW (7000 K), NW (4000 K) and WW (3000 K) FL: NW 3600 K Philips Master TL-D 58 W/835	Illuminance (lx): 5000 measured:center of the shelf. PPFD (μmol m^−2^ s^−1)^: FL 33.7 ± 11.4; LED 7000 K: 44.2 ± 15.3; LED 4000 K: 40.9 ± 13.7; LED 3000 K: 43.8 ± 14.8 GL Spectrolux spectrometer; GL Optic	Shoot length, growth, secondary metabolites production	LEDs results as a more efficient, eco-friendly and economically reasonable source of light for big scale in vitro production than FLs	[31]
*Morus spp*	LED (nm): R:G:B (635/520/452), R (635) and B (452). FL: W (C) Graph spectrum but not given definition values or charactersitics	PPFD (μmol m^−2^ s^−1^): 10 and 50 Spectral Colour Illumino meter; Hangzhou Hopoo	Deoxynojirimycin (DNJ) content	R LED particularly increased the DNJ production	[32]
*Schomburgkia crispa* Lindl.	LEDs: WW (3000 K) and CW (6500 K) FL: CW 6500 K [C]	PPFD LED (μmol m^–2^ s^–1^): WW 128; CW 58; and CW 108 PPFD FL (μmol m^–2^ s^–1^): CW FL 23	Germination %	3000 K LED induced a faster establishment and low mortality	[33]
*Dracocephalum forrestii*	LED (nm): B (430), R (670), B:R (7:3) and NW (430–670) -Graph spectrum but not given definition values- FL: CW [C] -undefined-	Spectrometer BTS256-LED Tester; Gigahertz-Optik PPFD (μmol m^–2^ s^–1^): 40	Shoot proliferation and length; FW; DW; photosynthetic pigments; secondary compounds with therapeutic value	W LED induced the best proliferation. B was optimum for biomass and micropropagation. LEDs increaseed secondary metabolites accumulation	[34]
*Hylocereus costaricensis*	LEDs (nm): CW (400–700) [C], MW LEDs (540), R (660), B (440) and R:B (660 and 440). Osram Opto Semiconductors Darkness FL: CW Phillips TLD, 36 W Graphs of relative spectral distribution given	PPFD (μmol m^–2^ s^–1^): CW 30, MW 17.5, R 37.9, B 18.5, R:B 61.1, CW FL 30 SPIC200 portable spectral irradiance colourimeter; Everfine Corporation	Betalain pigment, phenolic and flavonoid content, antioxidant activity	R enhanced betalain content in Y and R callus. B and R + B light improved antioxidant properties. R enhanced phenolics, flavonoids and antioxidant activity in R callus	[35]
*Pteris aspericaulis var. tricolor*	LEDs (nm): W (450–470, 570–590), B (440) and R (660)	PPFD (μmol m^−2^ s^−1)^: 40	GGB differentiation frequency and FW, plant height, leaf morphology	LEDs did not affected GGB multiplication and differentiation. R promoted elongation, but B inhibited it	[36]
*Ceratophyllum demersum*	LED: W -undefined-	Iluminance (lux): 1500	FW, DW, Cr uptake, BCF	In vitro plants are useful for Cr phytoremediation	[37]
*Vaccinium corymbosum* L.	LED: R:B (77:23) and W -undefined- FL: W -undefined-	PPFD (μmol m^−2^ s^−1^): 55 ± 12	No. of shoots, FW, DW	R:B LEDs induced more production of meristems and biomass than W LED or FLs	[38]
*Campomanesia pubescens (DC.)*	LED (nm): R (600–700), B (400–490), W (400–700) and R:B (1:1) -undefined-	PPFD (μmol m^−2^ s^−1)^: 50 ± 5 PPFD sensor QSO-S; Decagon Devices, Pullman	Biometry; leaf area and anatomy; MDA, DW; CHL, CNOC	R increased MDA, with oxidative damages. W, B:R enhanced biometry	[39]
*Lippia filifolia*	LED (nm): W (400–700) [C], B (450), R (653), R:B (664 and 448)	Spectrorad. R Tide USB 650 UV, Ocean Optics™	Height, shoots no., FW, CHL, CNOC, others	R:B enhanced the growth and the regeneration	[40]
*Passiflora edulis* Sims	LED (nm): Combination of B (450), G (525), R (660), and IR (730)	PPFD (μmol m^–2^ s^–1^): 42	Height, bud, root no., CHL content	R and mTR promoted a reliable propagation	[41]
*Solanum tuberosum* L.	LED: R (660), B (450), R:B (65:35) and R:B:G (520) (45:20:35) FL: W (C) -undefined-	PPFD (μmol m^–2^ s^–1^): 100	Stem, root length and Φ, health index, leaf area, FW, DW, starch, others	B, R:B, R:B:G LEDs were better than FLs for micropropagation	[42]
*Fritillaria cirrhosa* D. Don	LED (nm): R (660), B (450), fR (730), CW (5000 K), WW (2700 K) R:B (8:1), R:G (525):B (7:1:1) and R:B:fR (1:1:1)	PPFD (μmol m^–2^ s^–1^): CW 57; WW 56; R:G:B 56; R:B 57; B 57; R 56; fR 10; R:B:fR 56	No. of SE, FW, alkaloid content	R, fR increased alkaloid content. R, fR, R:B:fR enhanced FW and SE nº	[43]
*Bambusa oldhamii* Munro	LED (nm): B (455), R (630), R:B (30:70; 70:30) (TEC-LUX LED) FL: W (C) -undefined-	PPFD FL (μmol m^–2^ s^–1^): 40	Shoot height, no. of shoots and leaves	B:R, TDZ and gas exchange increased shoot proliferation	[44]
*Dysphania ambrosioides* L.	LED: B, R, W and B:R [1:1, 2:1, 1:2] -undefined-	PPFD (μmol m^–2^ s^–1^): LED: 42; FL CW: 60	Shoot length; leaf, shoot and root dry biomass; VCO	W, B:R [2:1] increased the growth. B reduced Z-ascaridole content	[45]
*Limnophila aromatica* & *Rotala rotundifolia*	LED: W, R, B, W:R:B (2:1:1), W:R:B (1:2:1), W:R:B (1:1:2) and W:R:B [1:1:1] -undefined- FL: W [C] -undefined-	Iluminance (lux): 1500 Luxometer PCE-EM 888	Regeneration %, no. and length of shoots	W:R:B [1:2:1] was the most effective for in vitro propagation	[46]
*Anoectochilus roxburghii*	LED (nm): R (630), B (465), B:R (20:80), B:R:W (13.8: 72.4: 13.8; 13.8: 58.6: 27.6) FL: (C) -undefined-	PPFD (μmol m^–2^ s^–1^): 30 ± 2.13 Distance between plants and lights: 40–60 cm	Stem diameter, leaf no. and area, height, FW, DW, root no., stem anatomy, flavonoids	BR [1:4] enhanced the flavonoids content, the propagation and the medicinal value	[47]
*Camellia oleífera* Huajin	LED (nm): R (640), B (450), R:B (4:1) and R:B (1:4) LED: W (C) -undefined-	PPFD (μmol m^–2^ s^–1^): 50 ± 5	Proliferation, length, height, CHL, CNOC, leaf anatomy, proteins	R:B [4:1] induced the highest proliferation coefficient	[48]
Brassica eruca, ‘Rocket’ and Brassica juncea, ‘Ruby Streaks’	LED (nm): R (665), B (440) (Pro-Series 325 by LumiGrow)	PPFD (μmol m^–2^ s^–1^): 20, 70, 120, 250, 450 and 650	Dry mass, hypocotyl and petiole elongation, size, plant coloring	B LED promoted elongation, but this varied with light intensity and plant species.	[49]
*Solanum tuberosum* L.	LED (nm): B (440), B (460), G (520), Y (590), R (620) and R (660)	PPFD (μmol m^–2^ s^–1^): 65	Height, stem diameter, leaf and microtuber no., CHO	Light reduced the production cycle, and increased microtubers	[50]
*Elaeis guineensis*	LED (nm): R (660), B (460) and R:B [3:1] (660 and 460) LED W: 10,000 K; FL: W 6500 K, Phillips T5 28 W; DARK		FW, leaf no., shoot height, roots	R:B induced the growth. R enhanced the rooting	[51]
*Solanum tuberosum* L.	LED (nm): Comb of UV-A (380) + UV-A (400) + B (450) + G (520) + R (660) + fR (735) FL: WW 2700 K (+C) DARK (-C)	PPFD LED max (μmol m^–2^ s^–1^): 1000 PPFD FL (μmol m^–2^ s^–1^): 2.8–4.6	Sprout length, growth vigour	R at low irradiances, reduced elongation; fR at hight ones, reduced it according to the cv.	[52]
*Saccharum officinarum* L.	R:B LEDs (Philips Green Power R/B 150 43 W) DR:W LEDs (Philips GP DR/W 150 33 W) FL: W (OSRAM Sylvania)	PPDF (μmol m^−2^ s^−1^): R:B 70, 120 and 200 ± 25; DR:W 70 and 120; W FL 200	Shoot height, leaf no., yellow leaf %, shoot and root no., root length, FW, DW, CHL	Increases in light intensity stimulated plant height and leaf nº, without negative effects	[53]
*Cedrela fissilis* Vell	LED: W:mB, W:mB:DR, W:mB:DR:fR -undefined- LED (nm): B (425–490), DR (620–700) and fR (700–740) FL [C] -undefined-		Length and no. of shoots, FW, DW	BA, WmBdR: enhanced FW, DW and length. Proteins were identified	[54]
*Rehmannia elata* N.E. Brown ex Prein	W LED (8000–10,000 K) RB:B:lR:R:DR:fR (5:10:10:35:35:5) LED (nm): RB (430), B (460), lR (610), R (630), DR (660), fR (730)	PPFD (μmol m^–2^ s^–1^): 40	Shoot %, axillary shoots no., leaf area and width, roots no. and length, CHL	PAR illumination and PGRs enhanced the regeneration	[55]
*Solanum tuberosum* L.	LED (nm): W (C) -undefined-; R (650), B (460), R:B (3:7), R:B (1:1) and R:B (7:3)	PPFD (μmol m^–2^ s^–1^): W 100, R and B 210	Height, stem diameter, branches and leaves no., leaf area, FW, DW, health index, pigments, starch, soluble proteins, sugars and phenolics, ROS and ascorbate content	R:B (3:7) was optimal for plant development and growth	[56]
*Physalis angulata*	Luminaire: Screen filtered by Polysack’s Cromatinet^®^ black photoconverter mesh with 50% shading with 6 lamps	LED (nm): B (450), R (660), B:R, G (525), Y (590) LED CW (7000 k)	O2, CO2, stem and root length, leaf area, nodal segments, CHL, CNOC, leaf anatomy	Filtered natural light, allowed photoautotrophic propagation. LEDs did not promote it	[57]
*Hordeum vulgare* L.	LED (nm): B (454.63), G (525.95), R (630.84) DARK in callus induction		Quantitative analysis of DNA methylation	The methylation depends on the light conditions	[58]
*Pfaffia glomerata*	LED (nm): R (665), B (440), R:B (1:1), R:B (1:3) and R:B (3:1)	PPFD (μmol m^−2^ s^−1^): 80	Stem and root length, leaf area, DW, CHL, CNOC	R and B LEDs enhanced biomass and 20E production	[59]
*Libidibia ferrea*	LED: R:B and W -undefined-	PPFD (μmol m^–2^ s^–1^): 31 ± 1	Shoots length and no., multiplication, buds no.	R:B stimulated the growth	[60]
*Corymbia. citriodora* × *C. torelliana* and *C. torelliana* × *C. citriodora*	LED: CW -undefined- LED (nm): R:B (450 and 660) FL: -undefined-		Shoots length, no.; vigor, oxidation, CHL, CNOC content	R:B LEDs and sucrose enhanced elongation	[1]
Hybrid *Corymbia* clones	LED: CW -undefined- LED (nm): R:B (450 and 660) FL: -undefined-		Length, no., vigor and oxidation of axillary shoots	R:B, BA and ninth subculture, enhanced multiplication	[61]
*Scutellaria baicalensis*	LED (nm): B (420–480), R (600–650), WW (400–800) DARK [C]	PPFD (µmol m^−2^ s^−1^): 1	Growth of callus, flavones content	Light increased the callus. B induced flavones content	[62]
*Solanum xanthocarpum*	LED (nm): B (460), G (510), R (660) and Y (570) LED W (400–700 nm) -undefined- DARK	PPFD (µmol m^−2^ s^−1^): 45–50	Flavonoids, phenolics, phytochemicals, antioxidant activity	W increased the biomass. B, enhanced phytochemicals and phenolics content	[63]
*Dendrobium* Enopi x *Dendrobium* Pink Lady	LED CW (400–700 nm) LED (nm): fR (730), R (660), G (530), B (440) and B:R	PPFD (µmol m^−2^ s^−1^): CW 4.6, 5.2 and 17.0; fR 1.1, 9.1 and 20.8; R 1.3, 15.4 and 29.3; G 0.8, 6.2 and 16.9; B 0.9, 6.7 and 15.7; B:R 2.0, 20.3 and 44.8	Phenolics content, secondary metabolites accumulation	B:R increased flavonoids. R FL pre-illumination reduced LED effects on metabolites production	[64]
*Oryza sativa* L. cv. Nipponbare	FL W and LED: B, B:R (3:1, 1:1; 1:3), R	PPFD (μmol m^–2^ s^–1^): 50	Differentiation and budding rate, healthy index, plant no., length, CNOC, CHL, others	B improved callus regeneration. B:R [1:1] enhanced rice factory seedling cultivation	[65]
*Cunninghamia lanceolata*	LED: R:B (88.9:11.1), R:B:P (80:10:10), R:B:P:G (72.7:9.1:9.1:9.1), R:B:G (12.7:3.9:83.4) [C]	PPFD (μmol·m^−2^ s^−1^): 20 & 30	Plant height and no., rooting %, root no. and length, area, CHL	R:B:P:G was the best for in vitro growth	[66]
*Lippia grata* Schauer	LED R:B [5:1;1:1] and R:G:B LED (nm): B (460), R (640) and G (530) FL: W -undefined-	Non-ventilation under FL PPFD (μmol m^–2^ s^–1^): 60	Sprouts no., roots, leaves, FW, hyperhydricity, height, sucrose, CHO and CNOC content	Hyperhydricity was reduced under R:B and sealing; W decreased it in leaves	[67]
*Carpesium triste* Maxim.	LED: CW -undefined-, LED (nm): R (621–710), B (450–475), R:B (1:1) (400–700 nm	PPFD (μmol m^–2^ s^–1^): 50	Shoot diameter and length, root length and no., FW, DW	R and B LEDs produced high-quality in vitro plants	[68]
*Moluccella laevis* L.	LED (nm): W 8000–10,000 K (400–700) and B (430):B (460):R (610):R (630):fR (730) (5:10:10:35:35:5) (LED lamps Commled Solutions)	PPFD (μmol m^–2^ s^–1^): 180 Portable LightMeter HD 2302.0 equipped with LP 471 PAR and LP471 UVA detectors (DeltaOhm)	Shoots and buds proliferation, axillary shoots no. and length, callus diameter, CHL	PGRs effect on shoot growth and development was stronger than the light infuence	[69]
Two tomato cvs: House Momotaro and Mini Carol	Eight-peak LED [C] (nm) R (625 and 660), B (420 and 450), G (520), fR (730), UV (390) and W (400–700 nm) -undefined-; DARK	PPFD (μmol m^–2^ s^–1^): 226–249	ASA, DHA, antioxidant enzymes, H_2_O_2_, oxidative parameters	High light intensity enhanced ASA content. Differences with B and R LEDs were observed	[70]
*Musa* spp. CV. Dwarf Cavendish	LED R:B (18:2) FL: W -undefined-	Iluminance (lux): 1000	Shoots no., length, FW, DW, CHO, CHL, CNOC	R:B LEDs improved in vitro propagation	[71]
*Drosera burmannii* Vahl and *D. indica* L.	LED (nm): W (400–700), B (425–500) and R (600–700) -undefined- DARK	Irradiance (W·m^2^): 61.5 Distance lamps and plants: 30 cm	Plumbagin content	B LED enhanced plumbagin level, being higher in aerial parts	[72]
*Gerbera jamesonii*	FL: W (C) -undefined- LED (nm); R:B (7:3) (670 and 430)	PPFD LED (µmol m^−2^ s^−1^): 40, 80 and 120 PPFD WFL (µmol m^−2^ s^−1^): 40 [C]	Multiplication, leaf no., morphometry, axillary shoots, height, FW, DW, CHL	R:B and BA improved growth. High radiation enhanced leaf features	[73]
*Solanum tuberosum* L.	LED (nm): B (450), G (530) and R (660) LED: WW [C] –undefined-	PPFD (µmol m^−2^ s^−1^): 100 Distance lamps and shelf: 60 cm	Height, diameter, FW, DW, leaf area and no., health index	B increased health index. Light quality induced DEGs patterns	[74]
Chickpeas	R LED -undefined- FL: (EN 12464-1 FLUORA)	PPFD (µmol m^−2^ s^−1^): 70	Transgenic shoots, grafting	LEDs and micro-injury improved transformation	[75]
*Lippia rotundifolia* Cham	LED: W, R, B, R:B (1:1; 2.5:1;1:2.5) FL: CW -undefined-	PPFD FL CW (µmol m^−2^ s^−1^): 20, 54, 78, 88 and 110 PPFD (µmol m^−2^ s^−1^): 42	Shoot length, leaf no., DW (shoot, leaf, root and total), CHL, CNOC	R:B and low intensity promoted high growth and pigments	[76]
*Lycium barbarum* L. (Goji Berry)	FL: W (400–700 nm) –undefined- R:B LED (630 and 460 nm)	PPFD W FL (µmol m^−2^ s^−1^): 36 PPFD LED (μmol m^−2^ s^−1^): 86	Buds %, shoot length and no., leaf nº	RB stimulated shoots length and the multiplication	[77]
*Hygrophila polysperma*	LED: W, R, B, R:B, R:W, B:W, R:B:W -undefined-		No. of shoots	W:R:B incremented the shoots nº	[78]
*Bixa orellana* L.	FL: CW 4200 K LED W -undefined- B:R -undefined-	PPFD (µmol m^−2^ s^−1^): 50, 150 and 200	Leaf stomatal density, no. and area, bixin, MDA, CNOC, CHL	B:R and FL enhanced bixin and pigments depending on the cv.	[79]
*Curculigo orchioides* Gaertn	LED (nm): B (470), R (630), B:R (1:1) FL: CW (300–700 nm) -undefined- [C].	PPFD (µmol m^−2^ s^−1^): 50	Germination, shoots, roots, FW, DW, leaf area and density, CHL, CNOC, others	B and BR improved the synseed growth. R reduced growth and germination	[80]
*Arnebia euchroma*	FL CW (420 nm LED (nm): UV (410–416) B (450–455), R (650–660), R:B:W (25:25:50) and W//DARK	PPFD (µmol m^−2^ s^−1^): 60	Germination rate, shoot no., FW, DW, naphthoquinones content	RBW, R enhanced growth. R increaded roots and dark the naphthoquinones content	[81]
*Solanum tuberosum* L.	LED (nm): R (660), B (450), R:B (80:20; 70:30;50:50) FL: CW -undefined-	PPFD (µmol m^−2^ s^−1^): 40	FW, height, CHL, soluble sugar content	R:B (70:30) was the best	[82]
*Fragaria x ananassa* cv. Festival	LED (nm): fR (735):R (640):G (510):B (450):WW (3000 K) (15:55:15:10:5) FL: CW (C) -undefined-	PPFD LED (µmol m^−2^ s^−1^): 25, 50, 75 and 100 PPFD FL (µmol m^−2^ s^−1^): 45	Survival rate, shoots no. and lenght, leaf area, height, root no., rooting %, FW, DW	PPFD of 75μmol m^–2^ s^–1^, R:fR:B:G:WW was suitable	[83]
*Fagonia indica*	LED (nm): W (380–780), B(380–560), G (480–670), Y (530–780), R (610–715) -undefined- FL: W -undefined-; DARK	PPFD (µmol m^−2^ s^−1^): 40–50	FW, DW, flavonoid and phenolic contents, callus, antioxidative enzyme activities	W LED enhanced phenolics and flavonoids production. Under B LED, SOD and POD were best	[84]
*Cariniana legalis* (Martius) O. Kuntze	LED W: -undefined- LED: low B and DR, W:low B, DR: fR, W: medium B: DR, W:medium B:DR FR -undefined- FL: (C) -undefined-	PPFD (µmol m^−2^ s^−1^): 55	Length and no. of shoots, shoot induction	B:R:fR light induced shoot elongation. W:B:R LED affected the endogenous contents of Pas	[85]
*Acacia melanoxylon*	W LED (400–700 nm) R:B LED (1:1; 1:4; 1:4) -undefined- FL: (C) -undefined-	PPFD (µmol m^−2^ s^−1^): W LED: 45, 90 and 135 LED and FL: 135	Bud proliferation, growth, rooting rate, length and no.	R:B promoted growth. High photoperiod and intensity enhanced growth and reduced the proliferation	[86]
*Pyrus communis* L.	LED (nm): B (454), R (660), fR (745), R:B (1:1), fR:B (1:1), R:fR (1:1) FL: WW 3000 K (C)	PPFD (µmol m^−2^ s^−1^): 40	No. and length of shoots, callus weight, leaf area, CHL, CNOC	R promoted shoots length; fR stimulated the nº, but reduced the shoot quality. B enhanced the callus growth	[87]
*Tulipa tarda* Stapf	FL: W (390–760 nm), R (647–770 nm), B (400–492 nm) DARK [C]		Frequency of differentiation, no. of adventitious bulbs, FW	Dark enhanced the adventitious bulbs nº. Light spectra did not produces differences	[88]
*Phalaenopsis* ‘Fmk02010’	LED: R, G, B, W, R:G, R:B, R:W G:B, G:W, B:W, R:G:B, R:B:W, R:G:W, G:B:W -all undefined- FL: W (C) -undefined-	PPFD (µmol m^−2^ s^−1^): 54	No. and FW of PLBs, shoots, and roots, length of shoots	R, B or R:B used first and then W enhanced the regeneration and specifics CHO content	[89]
*Gerbera jamesonii*	LED (nm): R (657), B (450), R:B (8:2; 7:3; 6:4; 5:5) FL: W (C) -undefined-	PPDF (µmol m^−2^ s^−1^): 40	Height; leaf length and no.; root length and no.; DW, CNOC, CHL	R:B (7:3) improved growth and photosynthetic activity	[90]
*Chrysanthemum × grandiflorum*, *Gerbera jamesonii*, *Heuchera × hybrida*, *Ficus benjamina*, and *Lamprocapnos spectabilis*	LED: B:G:R:FR (14:16:53:17; 12:19:61:8; 8:2:65:25) UV:B:G:R:FR (1:20:39:35:5) -undefined- FL: CW 6200 K (C)	PPFD FL (µmol m^−2^ s^−1^): 62–65	Micropropagation efficiency, shoot length, leaf and root no., root length, FW, DW, CHL	B:G:R:fR LED was the best for plant quality, micropropagation and cost reduction	[91]
*Ocimum basilicum*	LED (nm): R (660), B (460), G (510), Y (570), W (400–700) FL W (400–700 nm) (C)-undef- DARK	PPFD (µmol m^−2^ s^−1^): 40–50	Phenolic and flavonoid content	LEDs light is a potent elicitor for in vitro metabolites production	[92]
*Stevia rebaudiana* (Bertoni)	Solar Box (SB) LED LED: R:G:B, R:B LED WW (3000 K) FL: W 6500 K [C]	LED (nm): R (650), G (520), B (450) PPDF FL (µmol m^−2^ s^−1^): 49 PPDF LED (µmol m^−2^ s^−1^): 75, 135, 230 and 382	Height, internode and leaf length, leaf width, FW and DW of roots and shoots	Light intensity at 75 to 230 μmol m^−2^ s^−1^ improved the plant development	[93]
*Alpinia purpurata*	LED: -undefined- FL -undefined-	PPFD (µmol m^−2^ s^−1^): 72	Shoot and leaf no., shoot length	The light effect varied according to the cv.	[94]
*Paeonia ostii* Fengdan	LED: R, B, R:B –undefined- DARK (C)	PPFD (µmol m^−2^ s^−1^): 50	Hypocotyl and epicotyl dormancy breaking %	Dormancy was broken in dark, R or B depending on the case	[95]
*Bacopa monnieri* (L.) *Wettst*	LED (nm): B (425–500), R (600–700) LED: W (400–700) (C); DARK		Triterpenoid saponin glycosides content	B light was the most suitable for bioactive compound content	[96]
*Cunninghamia (C.) lanceolata*	R:B (4:1; 8:1), R:B:P (8:1:1), R:B:P:G (6:1:1:1; 8:1:1:1) W LED (C) -undefined-	LEDs (nm): Red (620–630), B (460–470), P (410–420), G (520–530) Illuminance (lux): 600–700 on the surface of the bottles	Rooting rate, root no., surface area and activity	R:B:P:G enhanced root growth, and R:B:P:G (8:1:1:1) was the best	[97]
*Agastache rugosa*	LED W: WL 2700 K; WL 3000 K, NW 4000 K LED (nm):RB (450), B (470), B (500), G (525), R (660), fR (720) FL: CW 4000 K	PPFD FL and WLED (μmol m^−2^ s^−1^): 40	Axillary bud breaking %, axillary shoots no., length of shoot	The age of cultures, light and amino acids affected the phenolic compounds content	[98]
*Punica granatum* L.	LED (nm): UV (<400 nm), B (400–500), G (500–600), R (600–700), fR (700–800)	PPFD (μmol m^−2^ s^−1^): 200 ± 20	Leaf no., leaf area, root length, shoot height, FW and DW of shoots, leaves and roots	FLs reduced the roots. B, R, high G, enhanced the morphological features	[99]
*Myrtus comutis* L.	LED (nm):B (430), R (670), R:B (70:30) FL: W 6200 K (C)	PPFD (μmol m^−2^ s^−1^): 35	Multiplication, height, leaf no., FW, DW, CHL, CNOC, others	R stimulated growth and polyphenols. BA enhanced growth	[100]
*Salvia miltiorrhiza*	12 light treatments. Combination of LED (nm): R (660), G (525), B (450), fR (730) and UV (380)		Tanshinone IIA (TSIIA) content	LEDs affected secondary metabolite production through gene regulation	[101]
*Solanum tuberosum*	LED (nm): R (660), B (440) and G (525) LED WW -undefined-	PPFD (μmol m^−2^ s^−1^): 75	Stem diameter, height, nodes no., leaf area, FW, DW	R:B stimulated the micropropagation and microtuber production	[102]
*Ajuga multiflora* Bunge	LED (nm): B (660), R (450) FL: CW -undefined-	PPFD (μmol m^−2^ s^−1^): 45	Shoots no. and length, tocopherols, CNOC	WFL and 2% sucrose increased shoots number. B, R enhanced the micropropagation	[103]
*Handroanthus ochraceus*	LED: WW (peaks 475, 550 nm) -undefined- FL: W (peaks 400, 440, 490, 550, 615, 710 nm) -undefined-	PPFD (μmol m^−2^ s^−1^): 15, 20, 30, 40, 50 and 60	Multiplication rate, hyper hydricity; roots %; shoots and roots FW, DW and length; leaf number	High-power LED irradiation increased the shoot growth	[104]
*Lippia gracilis*	LED -undefined-: R, B, R:B (2.5:1; 1:2.5) FL: CW –undefined-	PPFD (μmol m^−2^ s^−1^): 26, 51, 69, 94 and 130	Shoot and root length, shoot and leaf no., DW, CHL, CNOC	R and 94 µmol m^−2^ s^−1^ stimulated the growth. B, the photosynthetic pigments	[105]
*Solanum tuberosum* L.	LED (nm): R (630), B (445–465), Y (590), G (520), R:B:Y [6:2:1], R:B:G (6:2:1), R:Y:G (6:2:1), R:B:G (6:2:1) FL: (400–700 nm) (C) -undef-	PPFD (μmol m^−2^ s^−1^): 72 ± 2	Stem and root length, stem diameter, health index, leaf area, CHL, FW, DW, starch, others	R:B:Y LED increased the vigor in in vitro plants	[106]
*Brachypodium distachyon*	LED: CW -undefined- LED: B:R (450 and 660 nm) FL: CW -undefined-	PPFD FL (μmol m^−2^ s^−1^): 150 PPFD LED (μmol m^−2^ s^−1^): 56	Height, DW, shoot no., panicles, roots, histochemical analyses	Light quality regulates cell wall deposition and lignification patterns	[107]
*Dianches caryophyllus* L.	LED: R and B -undefined- FL: CW -undefined-	PPFD (μmol m^−2^ s^−1^): 50	Adventitious shoot, proteins levels	FLs increased hyperhydricity R and B LEDs reduced it	[108]
*Dianthus caryophyllus* L.	W LED [C] -undefined- LED: B, R, R:B -undefined-		Shoots no. and length, hyperhydricity, plant quality, CLH	B or R LEDs and ventilation improved the quality of plants	[109]
*Phalaenopsis* y *Cymbidium*	LED: R, B, R:B (90:10), R:B LED (80:20), R:B LED (70:30) -undef- FL: Growlux [C]	PPDF FL (μmol m^−2^ s^−1^): 45 PPDF LED (μmol m^−2^ s^−1^): 60–75	Leaf no, height, shoot and root FW, DW, root length and nº, CLH	R and B enhanced the in vitro propagation. R produced weak plants with thin stems	[110]
*Gerbera jamesonii*	LED (nm): R (430), B (670), R:B (50:50; 70:30), R:B:W (430–730) (40:40:20) and R:B:fR (730) (49:49:2) FL: CW 6200 K [C]	PPFD (μmol m^−2^ s^−1^): 40	Shoot no., plant length and heigh, rooting %, roots length and no., leaf no., DW, CLH, CNOC	R:B (70:30) incremented the multiplication. R was optimal in rhizogenesis	[111]
*Lilium regale*	LED (nm): R (670), B (430) and R:B (70:30). FL: CW 6200 K (C) DARK	PPFD (μmol m^−2^ s^−1^): 35	Regeneration %, bulbs, shoots and roots no.	B and B:R enhanced the organogenesis, dark and FL reduced it. R promoted roots, but reduced bulb growth	[112]
*Heliconia Champneiana* cv. Splash.	LED (nm): R (620–630), B (455–475) and R:B [70:30] FL: W (380–780 nm) [C] -undef-	PPFD (μmol m^−2^ s^−1^): 25	Height, FW, no. and length of roots, no. of leaves	B reduced the growth, improved the quality and the survival in acclimatization	[113]
*Campomanesia rufa*	FL: W (20 W) -undefined- LED: R:B (7:3) -undefined-	PPDF FL (μmol m^−2^ s^−1^): 44 PPDF LED (μmol m^−2^ s^−1^): 98	Shoots and buds no., shoots length, leaves no.	BAP and FL W FLs improved shoots and buds growth	[114]
*Corymbia. torelliana x C. citriodora*, *C. citriodora x C. torelliana*	LED (nm): B (450) and R (660) FL: Grolux DARK	PPFD (μmol m^−2^ s^−1^): 80	Shoots length and no., contamination, oxidation	R, B LEDs produced the best results in in vitro propagation	[115]
*Chrysanthemum morifolium Ramat* cv. Jimba	LED (nm): G (565), B (450), R (660), Y (590), B:R (10:90; 20:80; 30:70; 40:60; 50:50; 60:40) FL: (C) -undefined-	PPFD (μmol m^−2^ s^−1^): 40–45	FW, DW, leaf size, no. and stomata density, photosynthesis rate	Microponic system, R:B (70:30) and AgNPs enhanced the development	[116]
*Camillia sinensis*	LED: B, R, FR, W -undefined- FL: W (C) -undefined-	PPDF FL (μmol m^−2^ s^−1^): 30–40 PPDF LED (μmol m^−2^ s^−1^): 50	Leaf area and FW, stem length and diameter, CLH, CNOC	R increased growth. B stimulated CsLHY expression and fR inhibited	[117]

## Data Availability

Not applicable.

## Glossary

ASA: ascorbic acid	DHA: dehydroascorbic acid
B: blue	DR: deep reed
BA: 6-benzyladenine	DW: dry weight
BCF: bioconcentration factor	FLs: fluorescent lamps
C: control	fR: far red
CCC: chlorocholine chloride	FW: fresh weight
CHO: carbohydrate	G: Green
CHL: chlorophyll	HP-LED: high-power LED
CNOC: carotenoid	HPLC: high-performance liquid chromatography
CsLHY: gene	IAA: indole-3-acetic acid, an auxin
Cv: cultivar	IR: infrared
CW: cool White	LEDs: light emitting diodes
DEGs: differentially expressed genes	MDA: malondialdehyde
mTR: meta-topolin riboside	SE: somatic embryos
MW: mint white	SOD: superoxide dismutase
NW: neutral white	Spd: spermidine
P: purple	TSIIA: tanshinone IIA
PA: polyamine	V: violet
PAR: photosynthetically active radiation	VOCs: volatile organic compounds
Pas: free polyamines	W: white
PGRs: plant growth regulators	WW: warm white
PLBs: protocorm-like bodies	Y: yellow
POD: peroxidases	2iP: N6 -(2-isopentenyl) adenine
PPFD: photosynthetic photon fux density	R: red
Put: Putrescine	
